# Pathology and Treatment of Insanity

**Published:** 1900-07-07

**Authors:** 


### Pathology and Treatment of Insanity.

The attention of the medical profession in the State of New York has lately been directed towards a question which, some few years ago, was raised in a modified form in London—the question, that is, whether the existing arrangements for the custody and treatment of the insane are of such a kind as to afford security for the employment of the full resources of pathology and therapeutics on their behalf. On April 11th, 1889, Mr. Brudenell Carter, then a member of the London County Council, moved in that body a resolution that a committee should be appointed to inquire into the advantages which might be expected from the establishment, as a complement to the existing asylum system, of a hospital, with a visiting medical staff, for the study and curative treatment of insanity. He argued that, while the three great functions to which the nervous system is subservient—sensation, motion, and thought—are all liable to derangements produced by disease, the derangements of motion and of sensation had of recent years been so effectively studied as to be brought more under the control of treatment than had formerly been the case, but that the derangements of thought were scarcely better understood, and not appreciably better managed, than they were when Conolly and his fellow-workers had accomplished the abolition of mechanical restraints. He attributed the difference largely to the essential differences which subsist between an asylum and a hospital, and urged the application of the hospital system to the insane. The difference between the two was described as depending upon the fact that, in a hospital, the visiting staff are free from domestic responsibility, and have no other duties than the investigation and treatment of disease, whereas in an asylum the medical superintendent is largely an administrative officer, whose skill in the accomplishment of purely medical work might not impossibly suffer from the claims of other duties, and whose interests were weakened by division. The committee held several sittings, and took the evidence of a large number of medical men of the highest reputation, with the result that they urged upon the Council the establishment of such a hospital as the mover of the resolution had proposed. The Council as a whole, perhaps influenced to some extent by financial considerations, and probably still more by the inability of the members to understand the true nature of the issue submitted to them, decided against their committee; but the Asylums Committee not long afterwards were so far influenced by the report as to adopt one of its recommendations, that, namely, which dealt with the appointment of a pathologist, and the gentleman selected for this office has already done excellent work. But the time and energies of the medical superintendent are still much consumed by non-medical duties, and it may fairly be doubted whether the largest attainable amount of benefit will ever be derived from isolated work in pathology, which is not in close and constant relation to that of a highly-trained clinical physician. In the State of New York the same question has been brought into prominence by a dispute, carried on with some acerbity, between the Pathological Institute of the New York State Hospitals and the State Commission in Lunacy. The New York *Medical News* of June 16th contains a very energetic protest, signed by a large number of the leading neurologists of the country, and said to be daily receiving fresh adhesions, against the alleged attempt to "interfere with or subvert the scientific work of the Institute" by placing it "under the control and direction of superintendents," and thus "diverting the energies of this research centre into routine educational schemes." The protest has also been unanimously adopted by the Section on Nervous and Mental Diseases of the American Medical Association. An editorial article in the same journal speaks of "the blighting influence" of the Lunacy Commission, of which a Dr. Wise seems to be the head, and even goes so far as to declare that what this gentleman extols as "the modern treatment of the insane" is "only the application to insane people of the ordinary rules of hygiene, sanitation, and dietetics, together with the treatment of intercurrent medical and surgical diseases. Treatment of insanity—the condition which justifies the presence of the insane in the State Institution—there is none." Again, "until some knowledge of the pathology of insanity has been gained there is no possibility of any scientific treatment of it, and the only hope of obtaining this knowledge lies in the work of the New York Pathological Institute, or of similar institutions." This is almost precisely the language of the report of the Committee of the London County Council, and we can only hope that the American agitation on the question may lead to results beneficial to humanity.

